# Hashimoto's Encephalopathy Presenting with Unusual Behavioural Disturbances in an Adolescent Girl

**DOI:** 10.1155/2017/3494310

**Published:** 2017-05-21

**Authors:** Murugan Selvaraj Karthik, Kulothungan Nandhini, Viswanath Subashini, Ramasamy Balakrishnan

**Affiliations:** Department of Psychiatry, Sri Ramachandra Medical College & Research Institute, Sri Ramachandra University, Chennai, India

## Abstract

Hashimoto's encephalopathy (HE) is a rare autoimmune disorder with neurological and neuropsychiatric manifestations and elevated titres of anti-thyroid antibodies. Here we are reporting a case of HE in a 19-year-old girl who presented with seizure-like episodes, confusion, and behavioural disturbances with catatonic symptoms such as posturing, echopraxia, echolalia, and ambivalence. Patient did not respond to antipsychotics and anticonvulsants. On further investigation, patient was found to have high serum anti-TPO antibodies of about 1261 U/mL with euthyroid status, which supported a suspicion of HE. Our consultant neurologist confirmed the diagnosis and she was started on injection of methylprednisolone 750 mg OD. Since patient started showing clinical improvement, her antipsychotic medications were tapered off. On follow-up, patient has recovered and is functioning well. Since HE is a diagnosis of exclusion, very high anti-TPO antibodies and good response to steroids supported the diagnosis of HE in this patient after excluding other etiological possibilities. This case has been reported because the clinical presentation was predominantly neurobehavioural manifestations which is uncommon with HE.

## 1. Introduction

Hashimoto's encephalopathy (HE) is a rare neuroendocrine disorder that may be often misdiagnosed and mistreated for a considerable time. The common neurobehavioural manifestations include seizure-like episodes which are often resistant to anticonvulsive treatment, myoclonus, confusion, headaches, hallucinations, stroke-like episodes, coma, impairment of cognitive function, mood swings, focal neurological deficits, disturbance of consciousness, presenile dementia, psychosis, and ataxia [1, 2]. However, such manifestations are not common clinical presentation of HE.

HE is more common in women than in men and occurs in all age groups. The estimated prevalence of HE was 2.1/100,000 [3]. An Asian study summarized the findings of 105 cases of HE and reported that, in most cases, the diagnosis was based on altered consciousness, negative finding for infective aetiology in cerebrospinal fluid (CSF), and high level of thyroid antibodies, with the latter found in 100% of the cases. Moreover, a high protein level in the CSF appeared in 78% of cases and abnormal electroencephalography (EEG) in 98% of cases [4]. In another review, the magnetic resonance imaging (MRI) of brain revealed ischemic areas, multiple tumours, granulomas, or various degenerative processes in 60% of the cases and single-photon emission computed tomography (SPECT) examination showed decreased perfusion in the cortical areas or basal ganglia [5].

## 2. Case Report

A 19-year-old girl presented with complaints of three episodes which mimic seizures during sleep over the past 3 months, first episode was characterized by loss of consciousness, tonic-clonic movements of both upper and lower limb, upward rolling of eyeballs, and tongue bite with postictal confusion lasting for 10 minutes, and the subsequent episodes were characterized by tonic-clonic movements of both upper and lower limbs and chanting “om namashivaya,” followed by unresponsiveness lasting for 10 to 15 minutes. Patient would sleep after these episodes and did not have any memories of those episodes. Then, one morning while sleeping she developed myoclonic jerks and screamed out of her bed with acute confusion, psychomotor agitation, and irrelevant talk which lasted for three days and was then brought to psychiatry OPD. There was no history suggestive of fever, headache, vomiting, and focal neurological deficits during this period. Physical examination was unremarkable. On examination, patient was restless, disoriented, producing abnormal clicking sound, and not obeying verbal commands. Patient was admitted to psychiatric ward for diagnostic clarification and further management. Patient was started on oral benzodiazepines and her behaviour was closely monitored. Her sleep and communication remained poor and she required assistance for maintaining her personal hygiene. Routine lab examinations were within normal limits. Her EEG recording showed normal study, and ultrasound abdomen and MRI brain plain and contrast were reported to be normal. Over the next couple of days, she was noticed to be pacing around aimlessly and started exhibiting abnormal behaviours such as dancing and crawling like snake. These behaviours varied over the days and she developed posturing, echopraxia, echolalia, and ambivalence subsequently. For these symptoms patient was started on injectable lorazepam 6 mg per day in three divided doses. Since patient's status remained the same after few days, she was started with oral olanzapine 5 mg/day which was gradually increased to 15 mg/day. Meanwhile, patient was evaluated for the possibility of infective etiology, which was ruled out by neurophysician. The CSF analysis showed normal cell count, protein, and glucose levels. Thereafter patient was evaluated for autoimmune encephalopathies. On investigation, patient had high serum anti-TPO antibodies of 1261.4 U/mL (normal < 60) with euthyroid status and negative for other autoimmune encephalopathies including antibodies for voltage gated potassium channel, which supported a suspicion of Hashimoto's encephalopathy. Therefore, again neurophysician consultation was sought and started on methyl prednisolone 750 mg/day intravenously for 5 days, followed by oral prednisolone 45 mg/day. Thereafter, patient started showing significant improvement clinically. Corticosteroids were gradually tapered over a period of 4 months and her antipsychotic medications were tapered off. On follow-up, patient recovered symptomatically and started functioning well.

## 3. Discussion

In this case, patient was reported with acute onset of neurobehavioural symptoms. All possible infective aetiologies were ruled out by neuroimaging and CSF Analysis and with appropriate serological investigations. Therefore, patient was symptomatically managed with psychotropic medications, which did not produce any significant improvement in her clinical status and was further complicated by the development of catatonic symptoms. Patient was further investigated for other possible etiologies such as autoimmune encephalopathies, of which anti-TPO antibodies were found to be elevated in spite of euthyroid status. Several case reports and research evidence suggest that the presence of elevated serum levels of antithyroid antibodies remains an essential characteristic of HE diagnosis, despite normal thyroid hormone levels [6, 7]. However, the titre of anti-thyroid antibodies does not correlate with severity or improvement [8]. The mechanism of HE does not appear to be related to the thyroid status, which can vary greatly in patients with HE. Several studies in the past reported varying thyroid status in HE patients, about 23% to 35% of patients had subclinical hypothyroidism, 17 to 20% had hypothyroidism, 7% had hyperthyroidism, and 18% to 45% of patients were euthyroid [4, 7]. The closely related autoimmune disorder, limbic encephalitis, was ruled out with negative voltage gated potassium channel antibodies. However, another close differential diagnosis, which shows similar manifestations as HE, is anti-NMDAR encephalitis. In our opinion, patient with anti-NMDAR encephalitis might represent more protracted courses and poor outcomes. HE, in general, responds successfully to corticosteroid treatment, whereas about 50% of patients with anti-NMDAR encephalitis do not improve with first-line treatment such as corticosteroid, IVIG, and plasma exchange; thus, they should continue to have second-line immunotherapy using rituximab, cyclophosphamide, or both [9, 10]. Majority of patients with anti-NMDAR encephalitis have underlying neoplasms such as ovarian teratoma. Though few patients do not have an underlying neoplasm, in all those patients with neoplasm, removal of the underlying cancers with initiation of first-line therapy is the most important step in accelerating improvement and decreasing relapse [9]. Nevertheless, all patients should undergo extensive tumour screening, at presentation and at yearly intervals. Our patient underwent routine imaging examinations and found no tumours. Another unique finding indicating anti-NMDAR encephalitis is EEG record showing extreme delta brush (slow waves with overriding fast beta activity) in 30% of patients [11] which was in contrast to the normal EEG recording in our patient. Furthermore, we excluded anti-NMDAR encephalitis based on the clinical grounds. However, we were not able to correlate with laboratory findings due to logistic reasons which would have been more appropriate. Our case report demonstrates that high index of suspicion over HE is needed in the evaluation of patients with acute onset neurobehavioural clinical presentations.

## 4. Conclusion

Hashimoto's encephalopathy is a rare but very serious illness. The incidence is probably underestimated because of low overall awareness about the disease. HE may be found in cases of unexplained encephalopathy as well as in patients presenting with acute onset neurobehavioural manifestations, particularly together with the presence of high thyroid antibody levels, especially against thyroperoxidase. Likewise, even though steroid-resistant HE has been reported, other cause of encephalopathy should be considered when the response to steroid is not complete in cases with possible HE, especially in cases with any concomitant thyroid related diseases. Because of the autoimmune origin of the disease, corticosteroid treatment is usually proven to be effective. Therefore, HE needs to be added to the long list of differential diagnosis in cases presenting with atypical psychiatric presentation.

## Additional Points

*Limitation of the Study*. However, the major limitation of our case report is the lack of full antibody testing for NMDAR, LGi1, CASPR2, and other antibodies associated with autoimmune forms of encephalitis. Since HE is more of a diagnosis of exclusion, doctors should have a clinical suspicion and good knowledge of all other autoimmune encephalopathies and exclude them with appropriate serum markers before making a diagnosis of HE.
